# Exosome-Coated tPA/Catalase Nanoformulation for Thrombolytic Therapy

**DOI:** 10.3390/bioengineering10020177

**Published:** 2023-01-31

**Authors:** Sara Khalil, Mathumai Kanapathipillai

**Affiliations:** Department of Mechanical Engineering, University of Michigan-Dearborn, Dearborn, MI 48128, USA

**Keywords:** exosomes, nanoformulations, thrombolysis, tissue plasminogen activator

## Abstract

Current tissue plasminogen-based therapeutic strategies for stroke suffer from systemic side effects and poor efficacy. Hence, novel drug delivery methods are needed to overcome these shortcomings. Exosome-based drug formulations have been shown to have superior therapeutic outcomes compared to conventional systemic drug delivery approaches. In this paper, we report exosome surface-coated tissue plasminogen activator (tPA)/catalase nanoformulations with improved thrombolytic efficacy compared to free tPA, which also reduce side effects. The results showed that the tPA exosome formulations retained tPA activity, improved tPA stability, exhibited significant fibrinolysis, and showed no significant toxicity effects. Further, when combined with antioxidant enzyme catalase, the formulation was able to inhibit hydrogen peroxide-mediated oxidative stress and toxicity. Hence, exosome-based tPA/catalase nanoformulations could have the potential to offer a safer and effective thrombolytic therapy.

## 1. Introduction

Stroke is one of the leading causes of mortality in the United States and the world. Hence, finding an effective treatment is of great importance. Stroke can be categorized into ischemic and hemorrhagic stroke. The majority of strokes are ischemic and thrombotic in nature [1,2]. The onset of stroke often results in changes in blood physiology, including blood pressure, temperature, and altered blood oxygen and glucose levels [3]. Several changes in blood flow physiology after a stroke are often correlated with treatment outcome. Under normal conditions, various physiological responses, including cerebral perfusion pressure and cerebrovascular resistance, maintain normal cerebrovascular blood flow to the brain, and hence prevent cerebral ischemia [4]. However, during stroke, autoregulation is impaired, leading to abnormal flow and eventually ischemia. Since a higher probability of ischemic strokes occurs due to thrombosis, effective thrombolytic therapy is of importance for successful stroke treatment. The current FDA-approved thrombolytic drug is a tissue plasminogen activator (tPA). It binds to the fibrin in blood clots and activates plasminogen to form plasmin, which degrades the fibrin clots. However, it has been shown that tPA can cross the blood brain barrier (BBB), and it is associated with brain hemorrhage, causing severe brain damage [5,6,7,8,9,10]. It is estimated that only 5–7% of ischemic stroke patients receive intravenous tissue plasminogen activator (tPA), with another 1–2% receiving intra-arterial therapy [11,12].

Even when thrombolytic treatment is carried out within the initial couple of hours of the stroke onset, a cascade of events will result, depending on the nature of the initial thrombus. After several hours of stroke onset, due to poor blood and oxygen supply, there is an increase in reactive oxygen species (ROS) production, a breakdown of the BBB, and the infiltration of inflammatory signals and cells, all of which ultimately leads to severe neuronal damage [13,14]. Therefore, in addition to a thrombolytic drug, it is essential to address the ROS-related side effects. Apart from their negative side effects, balanced levels of ROS are needed for normal physiological functions and to inhibit mechanisms that lead to necrosis and apoptosis following an ischemic stroke. Several ROS inhibitory therapies have been studied. A catalase enzyme is one of the therapies that is used to overcome oxidative stress-mediated effects [15]. Catalase has been shown to mitigate oxidative stress and has been widely studied as an effective antioxidant in aging diseases including stroke [16,17,18,19]. Hence, it could be formulated with a thrombolytic drug for stroke treatment using an alternative approach to conventional systemic therapy that could reduce systemic side effects and significantly improve the therapeutic outcome of thrombolytic therapy.

Nanoformulations can be tuned to increase the therapeutic efficacy of a drug and have recently been used in several stroke treatment studies [20,21,22]. Korin et al. have shown tPA microaggregate formulations that could disintegrate into nanoparticles and exhibit enhanced clot lysis in a mouse pulmonary embolism model and in a mesenteric artery injury model [23]. In addition, the thrombolytic microaggregates, in combination with a stent retrieval therapy, exhibited enhanced recanalization in a rabbit carotid vessel occlusion model [24]. Uesugi et al. developed an ultrasound-responsive tPA nanodepot [25]. Gelatin was modified with cations and anions, and tPA was complexed with the cationic gelatin, and subsequently, a complexation was formed with anionic gelatin. The tPA was retained in the complex and released under ultrasound stimuli. In a rabbit thrombosis model, the nanocomplex facilitated complete recanalization. Enzyme-loaded nanoparticles have also shown promise in mitigating tPA-associated oxidative stress. Catalase and superoxide dismutase enzyme were encapsulated in PLGA nanoparticles, and when co-delivered with tPA in a rat thromboembolic model, neurological function was restored compared to rats treated with tPA alone [19]. Natural drug carriers have also been successfully studied in thrombolysis. Erythrocyte (red blood cell)-based thrombolytic drug delivery has been studied extensively [26]. Several nanomedicine strategies were employed to overcome hemorrhagic risk due to tPA. Erythrocyte-coupled tPA prevented cerebrovascular thrombosis and hemorrhage in a rat filament model of middle carotid occlusion [27]. In another study, it was shown that tPA genes packaged in albumin nanoparticles and crosslinked to microbubbles prevented thrombosis for up to two months without any adverse side effects [28]. Although several tPA nanoformulations have been studied over the years, the formulations lack efficacy and safety and hence still need improvement to be clinically relevant.

Compared to conventional nanoformulations, exosomes could be of particular interest due to their positive effect in stroke treatment. Exosomes are cell-secreted membrane vesicles that function in intracellular communication, whereby they deliver their content from one cell to the other. They are one of the promising nanobiomaterial-based drug delivery systems [29,30,31,32]. In addition to their nano-size, they have a slightly negative zeta potential that allows them to circulate for a long time in the body, their cytoskeleton is deformable, and some exosomes have the ability to escape the immune system, which they can circulate without being cleared from the body [33,34]. Although exosomes are synthesized under natural conditions at cellular levels, successful alterations are required to realize their full potential [35]. Moreover, exosomes extracted from different types of cells have different physicochemical properties and thus different pharmacokinetics [36]. Therefore, since these properties affect the therapeutic effectiveness of exosomes, it is important to properly select the cell from which the exosomes are extracted to be used in a drug delivery system [37,38,39,40]. After being isolated, exosomes require modification, depending on their structural properties and their basic cellular biology, in order to enhance their therapeutic and diagnostic abilities. Different small hydrophilic and hydrophobic molecules have been encapsulated in exosomes using different loading methods. Most experiments showed that delivering the drugs using exosomes leads to higher accumulation of drug at the targeted cells with an improved stability and a longer circulation time. It was also reported that exosomes could be used to carry therapeutic RNA, therapeutic proteins, and imaging agents that are hard to be delivered in vivo without a carrier [41,42].

Moreover, it has been shown that cell-derived exosomes showed positive outcomes in post-stroke therapy [41,43,44,45,46,47]. When an ischemic stroke occurs, brain cells, endothelial cells, and blood cells all release exosomes. Research has studied the changes that happen to the contents of exosomes, such as proteins and nucleic acids, during stroke [48]. Some studies have been conducted to evaluate the changes that occur to the exosomal contents during circulation, such as proteins and nucleic acid, and it was suggested that when stroke occurs, the exosomal profile changes, whereby some of these changes could worsen the stroke and increase the risk of other strokes, whereas others could be helpful as diagnostic tools and in recovery. For instance, some studies have shown the presence of certain exosomal miRNA in patients with strokes, suggesting that this could be used as a potential marker for diagnosing ischemic stroke and distinguishing between its different phases [44,48]. Other studies have shown that some exosomal miRNA play a role in protecting neurons against apoptosis [49,50]. The current trends and future directions of exosome-based stroke therapies have been published in detail by Schuldt et.al. [51]. Due to their signaling molecules and antimitogenic properties, exomes have emerged as a potential stroke therapy. Exosomes loaded with micro-RNAs, growth factors, small molecules, or surface functionalized with targeting moieties have been studied for the treatment of stroke [48,52,53]. However, exosomes modified with tPA and/or catalase have not yet been explored. Therefore, combining exosomes’ inherent therapeutic effects with tPA and catalase could prove an added advantage for stroke therapy compared to conventional methods.

In this study, we used exosomes surface-conjugated with the enzymes tPA/catalase to treat thrombosis. The exosomes’ tPA/catalase nanoformulations showed increased stability compared to free tPA. Further, formulations retained significant tPA activity in the presence of plasminogen inhibitor, compared to free tPA, and they exhibited significant fibrin clot lysis. In addition, the formulations did not exhibit toxicity or ROS to brain endothelial cells at the tested concentrations used in the study. Moreover, when treated with H_2_O_2_, the exosome-coated tPA/catalase formulation was able to significantly inhibit toxicity and oxidative stress effects compared to free tPA. The promising thrombolytic formulation could pave the way for exosome-based thrombolytic therapy for ischemic stroke. A description of the experimental methods and results is reported in the coming sections in detail.

## 2. Materials and Methods

Fibrinogen, plasminogen, and thrombin were purchased from Innovative Research, Novi, MI, USA. Alteplase (tPA) was purchased from the University of Michigan’s pharmacy. All the other chemicals were purchased from Millipore Sigma, St. Louis, MO, USA, or Thermo Fisher Scientific.

### 2.1. Exosomes tPA Conjugation

Exosomes were derived from human brain microvascular endothelial cells (hBMVEC) using ExoQuick-TC ULTRA kit (Systems Biosciences) according to the protocol. Exosomes’ surface markers, size, morphology, and concentration were characterized using flow cytometry, DLS, TEM, and protein assays, respectively. Next, exosomes were surface modified with EDC/NHS chemistry and subsequently conjugated with tPA. For the conjugation reaction, 200 μL of 50 μg/mL exosomes was reacted with 2 μL of 100 mM EDC and 2 μL of 100 mM NHS for 20 min, and subsequently, 20 μL (1 mg/mL) of tpA was added and stirred overnight at 4 °C. The exosomes were then centrifuged and resuspended in 220 μL HEPES buffer with a pH of 7.5. We used HEPES buffer for the reactions, as it has been widely used in tPA formulations [54,55,56]. To quantify the tPA conjugation to the exosomes, FITC (fluorescein isothiocyanate) or rhodamine-labeled tPA was used for the exosomes’ conjugation and quantified by the FITC or rhodamine fluorescence using an M3 SpectraMax plate reader. The particles’ size, morphology, and tPA protein conjugation were characterized by DLS, TEM, protein assay, and tPA activity assay.

### 2.2. Exosomes tPA/Catalase Conjugation

For the exosomes’ surface coating with tPA and catalase, a mixture of 40 μL (2 mg/mL) of catalase, 200 μL of 50 μg/mL exosomes, 2 μL of 100 mM EDC, and 2 μL of 100 mM NHS were reacted overnight at 4 °C. The particles were purified by centrifugation and then resuspended in 240 μL HEPES pH 7.5. Physicochemical characterization and efficacy studies were performed. A protein assay and a tPA activity assay were used to determine the catalase and tPA amount after conjugation.

### 2.3. TEM and DLS

Exosomes’ size and morphology were characterized by transmission electron microscope (TEM) and dynamic light scattering (DLS). Exosomes were diluted to a concentration of 5 μg/mL, and the size measurement was performed in three replicates. Transmission electron microscopy (TEM) images were obtained using a JEOL TEM at the Microscopy and Imaging Facility at the University of Michigan Medical School. Samples were placed on holey carbon cu grids, stained with 2% phosphotungstic acid, and imaged at 80 kV.

### 2.4. tPA Activity Assay and Protein Assay

The activity of tPA-coated exosomes was measured using a fluorometric tPA activity assay (Bachem, Fremont, CA, USA). Briefly, tPA and exosome-conjugated tPA were incubated with 0.5 mM of tPA substrate, and the activity was measured due to the cleavage of the fluorogenic substrate by tPA at 370/442 nm. The activity was compared with and without plasminogen activator inhibitor at an equimolar concentration of tPA. Measurements were carried out using a SpectraMax M3 plate reader. 

The protein assay was used to measure the concentration of exosomes, and to determine the conjugation efficacy of catalase and tPA. The protein concentrations were quantified by absorption at 562 nm according to the manufacturer’s protocol. The assays were performed at least three times.

### 2.5. Fibrin Clot Lysis Assay

For the fibrin clot lysis assay, fibrin clots were formed either in microcentrifuge tubes or 96-well plates. Clots were prepared by mixing 480 μL of 1 mg/mL fibrinogen, 6 μL of 1 M CaCl_2_, 2 μL rhodamine-NHS, and 120 μL of 100 units/mL thrombin at 37 °C for 24 h, after which the clots were washed by phosphate buffer and treated with tPA and thrombolytic exosome particles. After 2 h and/or 24 h of incubation at 37 °C, the fibrin clot lysis was measured by the rhodamine release using a SpectraMax M3 plate reader. At least three independent experiments were carried out for each condition.

### 2.6. Toxicity

To test the toxicity effects of the formulations to normal cells, human brain microvascular endothelial cells (hBMVECs) were plated on 96-well plates at a density of 2 × 10^4^ cells/well overnight in medium containing M-199, 10% FBS, and 5% Pen-Strep. The hBMVECs were obtained from Dr. Kalyan Kondapalli (University of Michigan-Dearborn) [57]. The details of the cell line are described in [58]. Cells were treated with, tPA, exosomes, exosomes-tPA, and exosomes-tPA/catalase, with and without H_2_O_2_ for 48 h. The concentrations used were 1 μg/ mL and 2 μg/mL tPA; 10 μg/mL and 20 μg/mL exosomes; 25 μM catalase; and 500 µM and 1000 µM H_2_O_2_. After 48 h, Alamar Blue was added. After 2 h of incubation, toxicity was analyzed by the fluorescent dye indicator resazurin at 570/590 nm excitation/emission using an M3 SpectraMax spectrophotometer in the lab. Three independent experiments were performed and analyzed.

### 2.7. ROS

The H2DCFDA assay (Thermo Fisher Scientific) was used to measure the oxidative stress associated with an increase in the production of reactive oxygen species caused by the treatment of tPA, exosomes, exosomes-tPA, catalase, and exosomes-catalase with and without H_2_O_2_ to brain endothelial cells. 2,7-dichlorodihydrofluorescein (DCFH)-based fluorescent probes have been widely used for oxidative stress quantification [59]. First, cells were cultured in 96-well plates at a cell density of 10,000 cells/well for 24 h, before being treated with the desired concentrations of the abovementioned conditions. After 24 h of treating the cells, the media was replaced by H2DCFDA for 30 min, and then ROS were analyzed by measuring the fluorescence at 495/526 nm excitation and emission. Cells were treated with the following concentrations: 1 μg/mL and 2 μg/mL tPA; 10 μg/mL and 20 μg/mL exosomes; 25 μM catalase; 500 μM or 1000 μM H_2_O_2_. Experiments were repeated at least 3 times and analyzed.

### 2.8. Permeability

To test whether the exosome-coated thrombolytic particles are transported through the blood brain barrier, an in vitro transwell model was used. hBMVECs at a density of 100,000/well were cultured on the upper chamber for 8 days to allow for tight junction formation, as previously described [22]. Then, exosomes were added for 2 h on the upper chamber. Transported exosomes were measured using FITC fluorescence. The percentage of exosomes extravasated into the lower chamber compared to that in the upper chamber at the beginning of the permeability experiment was quantified. Experiments were repeated three times to obtain statistical significance.

### 2.9. Statistical Analysis

Each experiment was repeated at least three times. The data were analyzed and presented as the mean and standard error of the mean (SEM). *p*-values were calculated from three or more independent experiments. For statistical significance, analysis of variance (ANOVA) followed by unpaired student t-tests was used. The significance of the results was presented as: ns: non-significant, * *p* < 0.05, ** *p* < 0.01, *** *p* < 0.001, **** *p* < 0.0001.

## 3. Results

**Figure 1:**
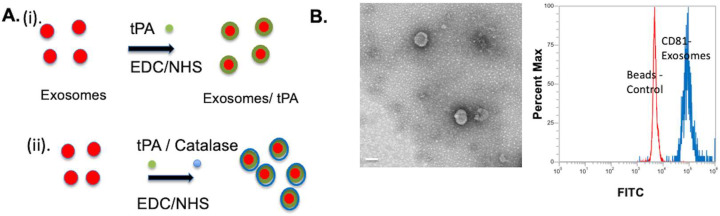
(**A**) Schematic of the exosomes’ formulation. Exosomes were first treated with EDC/NHS and subsequently reacted with tPA and/or catalase. (i). Exosomes conjugation with tPA, and (ii) Exosomes conjugation with tPA/catalase. (**B**) TEM image of the exosome and flow cytometry characterization of exosome marker CD81. Scale bar 100 nm.

For the study, exosomes were extracted from human brain microvascular endothelial cells and subsequently surface modified with tPA and/or catalase, as shown in the schematic in Figure 1A. Extracted exosomes were characterized by TEM, flow cytometry, DLS, and protein assay. The morphology and size of the exosomes were characterized with TEM images, which indicates sizes in the range of 170 nm (Figure 1B). The size was also characterized by DLS measurements, which revealed the exosomes’ size to be around 179 ± 25.875 nm (Table 1). To ensure that the exosomes were being extracted properly, we used flow cytometry as an additional characterization method to characterize exosome surface markers. The exosomes’ surface marker CD81 was characterized by flow cytometer. As can be seen, the retention of the surface marker was observed in exosomes’ conjugated beads. Next, the concentration of exosomes was determined by BCA protein assay. Exosomes extracted from 5 mL tissue culture media yielded around 250 μg/mL, and then concentrations at 50 μg/mL were used for the study.

**Figure 2:**
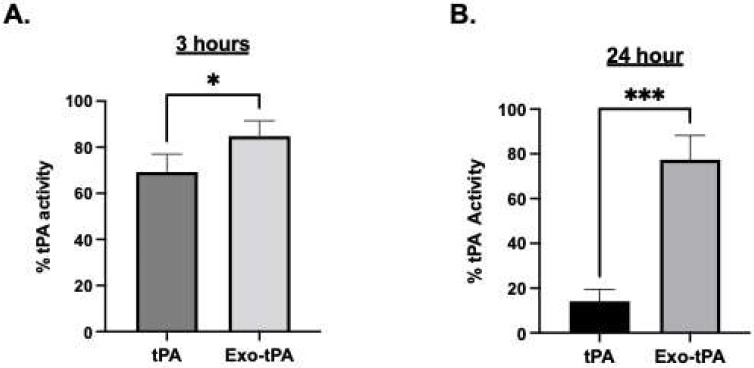
(**A**) Stability of tPA, and exo-tPA after 3 h. (**B**) Stability of tPA, and exo-tPA after 24 h. Stability of the exo-tPA, and tPA was measured using tPA activity assay. *n* = 3 ± SEM, * *p* < 0.05, *** *p* < 0.001.

Next, the exosomes were conjugated with tPA with and without a catalase enzyme. The conjugation of the enzymes increases the average size; exosome/tPA (exo-tPA) was found to be 317 ± 76.782 nm, and exosome/tPA/catalase was around 438.9 ± 10.712 nm, respectively, as shown in Table 1. The increase in the exosomes’ size is expected with tPA and catalase surface coating. The conjugation efficiency of tPA to the exosomes was found using FITC or rhodamine-labeled tPA, and it was revealed to be around 14.5 ± 4.2% from the calibration curve obtained for tPA fluorescence. The efficacy of the formulations was then tested by activity assay and fibrin clot lysis assay. To assess whether the exosome-conjugated tPA retained the enzyme activity, a fluorometric tPA substrate (Bachem, Fremont, CA, USA) cleavage assay was used, and the amount of cleavage was monitored at 370 nm/440 nm excitation/emission. The tPA activity in the exosomes was determined from the calibration curve obtained from free tPA activity under similar conditions. The stability of the exo-tPA formulation was assessed next. The exo-tPA formulation exhibited significant stability compared to free tPA over 24 h determined by tPA activity assay. As shown in Figure 2, the exo-tPA formulation increased the stability of tPA compared to free tPA at 37 °C. In particular, after 24 h, only less than 20% of free tPA was shown to be active compared to exo-tPA, which exhibited more than 75% activity.

**Figure 3:**
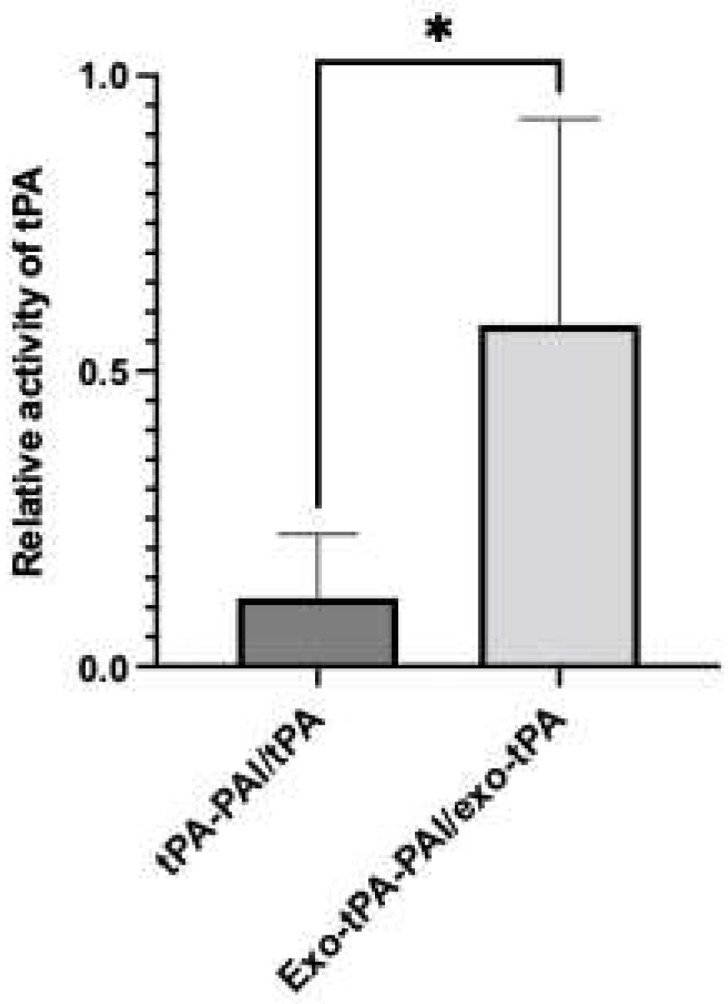
Activity of the tPA and exo-tPA with and without plasminogen inhibitor at equimolar amount. The tPA activity was assessed after 30 min treatment with plasminogen inhibitor at room temperature. *n* = 3 ± SEM, * *p* < 0.05.

**Figure 4:**
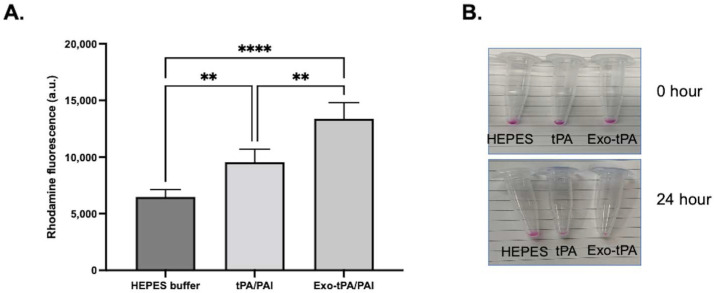
Clot lysis facilitated by exo-tPA and free tPA with equimolar amount of plasminogen inhibitor and plasminogen. (**A**). Clot lysis due to tPA, exo-tPA, and buffer treatments were measured at 24 h by the release of rhodamine from the clots. (**B**). Clot images at 0 h and 24 h after the treatment. *n* = 3 ± SEM, ** *p* < 0.01, **** *p* < 0.0001.

Next, we tested the activity of the exo-tPA formulation. The tPA activity assay was performed with and without plasminogen activator inhibitor (PAI) at an equimolar ratio, to determine the effect of PAI on free tPA and exo-tPA. tPA and exo-tPA samples were treated with and without the PAI at an equal molar ratio. As can be seen from Figure 3, the exo-tPA formulation retained its activity significantly compared to tPA alone. The results showed that the exo-tPA retained significantly more activity (70 ± 11.7%) compared to that of free tPA (13 ± 5.8%) in the presence of PAI. The results show that the activity of tPA when coated within the exosomes was not affected by the PAI. Meanwhile, the activity of the free tPA was affected, indicating the potential stability of the formulation under in vivo conditions. To determine the effectiveness of exo-tPA on its thrombolytic potential, a fibrin clot lysis assay was performed. Fibrin clots with rhodamine were prepared, and the desired concentration of tPA or exo-tPA, along with PAI and plasminogen, were added to the clots with a 1:1:1 molar ratio, with the concentrations being calculated based on tPA activity assays. The release of the rhodamine, indicative of fibrin clot lysis due to tPA activity, was measured 24 h after treatment. The tPA facilitates the activation of plasminogen to plasmin, which in turn drives the fibrinolysis. The rhodamine release from the clots due to the treatment of buffer, tPA/PAI, and exo-tPA/PAI were then plotted (Figure 4A). The corresponding clot images are shown in Figure 4B. Exo-tPA showed significantly better clot lysis efficacy compared to free tPA in the presence of PAI after 24 h of treatment, measured by the rhodamine fluorescence (arbitrary unit). No significant difference in clot lysis was observed between buffer treatment and tPA alone. The results suggest that exo-tPA has significant thrombolytic potential over free tPA in the presence of PAI.

**Figure 5:**
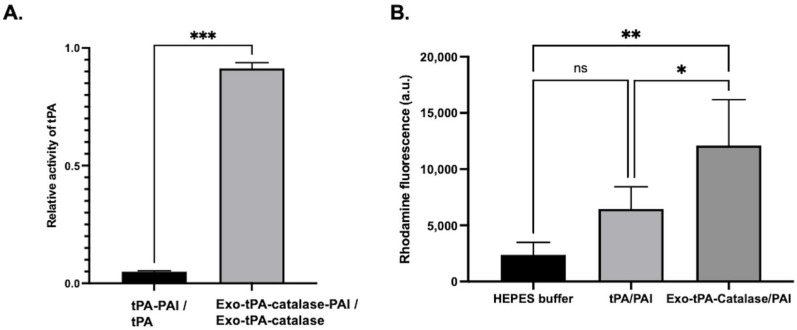
Activity and clot lysis assay of the exo-tPA-catalase formulation. (**A**) Activity of tPA and exo-tPA-catalase with and without plasminogen activator at equimolar amount. (**B**) Clot lysis due to tPA, exo-tPA-catalase, and buffer treatments were measured at 24 h. The rhodamine re-lease was measured using a plate reader. *n* = 3 ± SEM, * *p* < 0.05, ** *p* < 0.01, *** *p* < 0.001.

Next, we performed characterization and efficacy studies of the multi-enzyme formulation. The amount of catalase and tPA was determined by protein assay and activity assay. After preparing the exo-catalase and exo-catalase particles, we measured the catalase conjugation in the particles, using a BCA protein assay, to determine how much catalase was encapsulated within the exosomes. The results of more than three independent experiments showed that 30% of the added catalase reacted. The results show that the activity of tPA, when encapsulated within the exosomes, was not affected by the PAI, even in the presence of catalase. Meanwhile, the activity the free tPA was affected. From Figure 5A, when comparing the activity of tPA in both free tPA and exo-catalase-tPA, it was around 5% in the case of free tPA, whereas it was over 90% in exo-catalase-tPA. Then, we tested whether the multi-enzyme formulation was able to preserve thrombolytic activity. As from Figure 5B, the exosome/tPA/catalase formulation was able to induce significant clot lysis compared to free tPA or buffer treatment, indicating the retention of the activity. This shows that the tPA in exo-catalase-tPA successfully activates plasminogen, even in the presence of PAI, and breaks a fibrin clot effectively, whereas, when presented alone, the activation of plasminogen by tPA is affected by PAI and thus does not significantly dissolve the fibrin clot.

**Figure 6:**
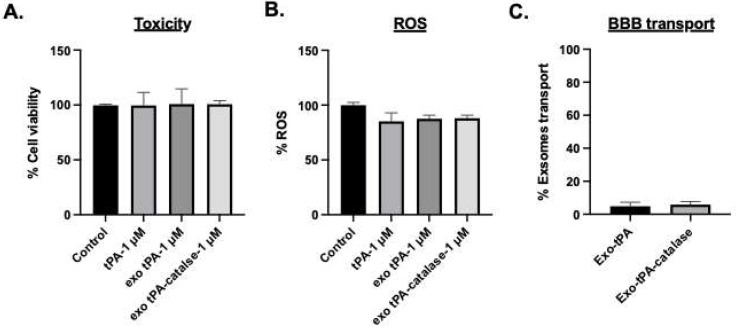
(**A**,**B**) Exo-tPA and exo-tPA-catalase toxicity and ROS to normal cells were assessed with Alamar Blue and DCFH-DA assay. *n* = 3 ± SEM. (**C**) Vascular transport of exosomes. Exosomes’ permeability was quantified by comparing the initial exosomes’ concentration at the apical side.

We then tested whether the formulations exhibit toxicity to normal brain endothelial cells. After 48 h of treatment with 1 μM of tPA or exo- tPA, exo-tPA-catalase toxicity was assessed. As can be seen from Figure 6A, the exo-tPA and exo-tPA-catalase formulations showed no significant toxicity. A similar experiment setup was then used to test whether the particle induces oxidative stress. The formulations did not exhibit oxidative stress (Figure 6B); none of the added materials exhibited ROS on the cells. In addition, we tested the transport of exosomes’ formulation across brain endothelial cells to assess the potential of the formulation to reduce hemorrhage. From the transwell assay study, the results showed that only about 4% of the exosomes extravasate (Figure 6C), indicating minimal damage to the brain microenvironment.

**Figure 7:**
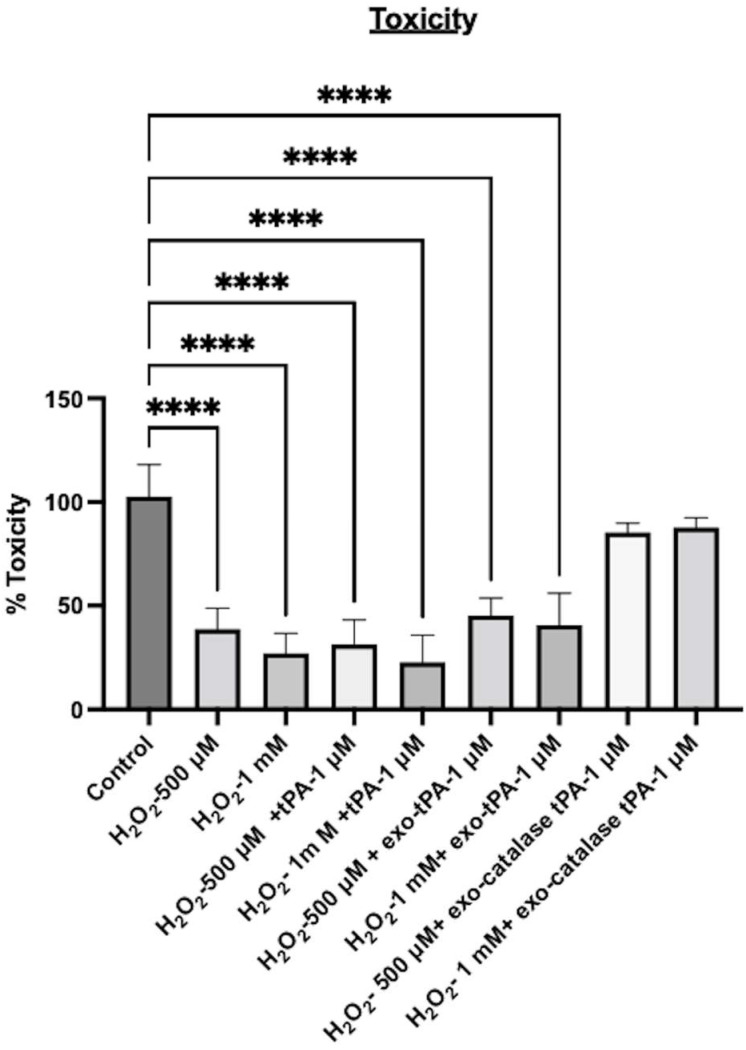
H_2_O_2-_induced ROS was assessed with DCFH-DA assay. Cells were treated with tPA or exo-tPA or exo-tPA-catalase with 500 µM or 1 mM H_2_O_2_. *n* = 3 ± SEM, **** *p* < 0.0001.

**Figure 8:**
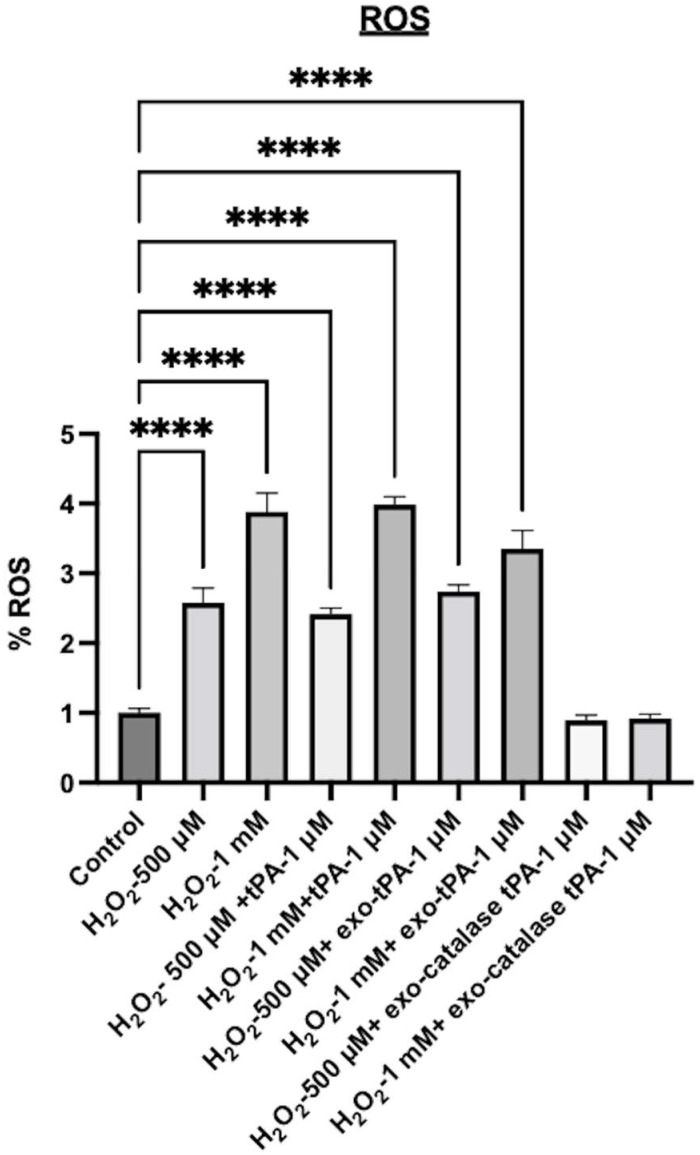
Human brain endothelial cell permeability of the exosomes was assessed in a transwell cell culture model. Cells were cultured for 9 days, and subsequently exosomes, exo-tPA, and exo-tPA-catalase were incubated for 15 min, and the amount of exosomes transported was compared to the initial amount incubated. *n* = 3 ± SEM, **** *p* <0.0001.

Next, to mimic post-stroke conditions, we tested the ability of the formulations to inhibit oxidative stress in the presence of H_2_O_2_. The tPA alone exhibited significant toxicity and ROS after the H_2_O_2_ treatment. Cell viability was 38% when treated with 500 μM H_2_O_2_ and 27% in the case of 1mM H_2_O_2_, and a similar range of cell viability was observed when tPA was added to the cells in addition to H_2_O_2_. Further, the tPA exosome formulation alone also did not prevent H_2_O_2_-induced toxicity. As can be seen from Figure 7, significant toxicity and ROS were observed with tPA exosomes. However, the tPA/catalase multi-enzyme formulation significantly inhibited the toxicity effects as well as the oxidative stress effects. The cell viability was above 80%, and the ROS was almost same as the control cells. When treated with exo-tPA-catalase, no significant cellular toxicity was observed, where cell viability above 85% was observed. Next, to determine whether catalase, tPA, H_2_O_2_, exo-catalase, and exo-catalase-tPA induce an increase in the amount of ROS released by the cells, an H2DCFDA assay was performed. The results showed that H_2_O_2,_ when present alone or with tPA or exo-tPA, exhibited significant ROS. However, exo-tPA-catalase was able to significantly inhibit H_2_O_2-_induced ROS (Figure 8).

## 4. Discussion

In this study, we have shown the potential of exosome-based tPA and exosome-based tPA/catalase formulations as therapeutics for thrombolytic stroke. The results show that the exosome-based formulation increases the stability and efficacy of the tPA in the presence of PAI. Further, when combined with catalase, significant inhibition of toxicity and ROS was observed. The efficacy of the formulation could be improved by further optimizing the reaction conditions. In this paper, we focused on basic in vitro studies to assess the potential of exosome-based thrombolytic formulation. To fully assess the potential of the exosome formulations and the efficacy of exosomes on blood clot lysis, studies on exosomes’ interaction with platelets, red blood cells, and protein adsorption should be studied in detail. A biocompatibility assay is an important assay to test the toxicity of the nanomaterial formulation [60]. In addition, platelet adhesion and protein adsorption assays are important to make sure the formulation does not promote thrombosis [61]. For future studies, we aim to perform hemolysis experiments, as mentioned by [60,61]. Further, the efficacy in animal models should be tested in future studies. Although several studies have reported thrombolytic nanoparticles for treating stroke [62], to our best knowledge, exosome-based multidrug formulations have not been studied before. Previous studies have shown that tPA delivery followed by catalase nanoparticles promoted post-stroke therapy [19]. However, systemic tPA delivery is not ideal, due to proven hemorrhagic side effects. Further, compared to conventional nanoparticles, exosomes have an added advantage due to their inherent therapeutic properties. The findings reported in this manuscript indicate that a multi-enzyme exosome formulation could lead to an effective thrombolytic therapy. 

As far as future directions are concerned, the current formulations still need vast improvement to realize their full potential in treating ischemic stroke. Exosome size, clot specificity, diffusivity inside the clot, fibrinolysis, and bioavailability need to be engineered for optimal exosome formulation. Once the formulation is optimized, in vivo animal studies should be performed to determine the clinical potential. Further, novel thrombolytic strategies, such as formulations for direct thrombolysis using plasmin and to minimize hemorrhagic side effects, or exosomes with novel multi-functionality or a combination therapy could have better outcome. The source of exosomes also needs to be investigated, since different cell sources have their own advantages and disadvantages. Alternatively, exosome mimetics could also be an attractive alternative for natural exosomes. Engineered exosomes have an added advantage compared to natural exosomes due to the tunable nature of the formulation for a particular application. Since stroke is one of the leading causes of death in the world, an improvement in therapeutic strategies and treatment modalities would be of paramount importance. Hence, continuous innovative nanotherapy research could play a major role in successful stroke treatment.

## Figures and Tables

**Table 1:** DLS measurement.

Nanoformulation	Size
Exosomes	179 ± 25.875
Exosomes + tPA (exo-tPA)	317 ± 76.782
Exosomes + tPA + catalase	438.9 ± 10.712
(exo-tPA-catalase)	

## Data Availability

Not applicable.
